# The zebrafish miR-125c is induced under hypoxic stress *via* hypoxia-inducible factor 1α and functions in cellular adaptations and embryogenesis

**DOI:** 10.18632/oncotarget.17994

**Published:** 2017-05-18

**Authors:** Yan He, Chun-Xiao Huang, Nan Chen, Meng Wu, Yan Huang, Hong Liu, Rong Tang, Wei-Min Wang, Huan-Ling Wang

**Affiliations:** ^1^ Key Laboratory of Freshwater Animal Breeding, Key Laboratory of Agricultural Animal Genetics, Breeding and Reproduction, Ministry of Education, College of Fishery, Huazhong Agricultural University, 430070, Wuhan, PR China; ^2^ Freshwater Aquaculture Collaborative Innovation Center of Hubei Province, 430070, Wuhan, PR China

**Keywords:** hypoxia, miR-125c, cdc25a, cell cycle, embryogenesis

## Abstract

Hypoxia is a unique environmental stress. Hypoxia inducible factor-lα (HIF-lα) is a major transcriptional regulator of cellular adaptations to hypoxic stress. MicroRNAs (miRNAs) as posttranscriptional gene expression regulators occupy a crucial role in cell survival under low-oxygen environment. Previous evidences suggested that miR-125c is involved in hypoxia adaptation, but its precise biological roles and the regulatory mechanism underlying hypoxic responses remain unknown. The present study showed that zebrafish miR-125c is upregulated by hypoxia in a Hif-lα-mediated manner *in vitro* and *in vivo*. Dual-luciferase assay revealed that cdc25a is a novel target of miR-125c. An inverse correlation between miR-125c and cdc25a was further confirmed *in vivo*, suggesting miR-125c as a crucial physiological inhibitor of cdc25a which responds to cellular hypoxia. Overexpression of miR-125c suppressed cell proliferation, led to cell cycle arrest at the G1 phase in ZF4 cells and induced apoptotic responses during embryo development. More importantly, miR-125c overexpression resulted in severe malformation and reduction of motility during zebrafish embryonic development. Taken together, we conclude that miR-125c plays a pivotal role in cellular adaptations to hypoxic stress at least in part through the Hif-1α/miR-125c/cdc25a signaling and has great impact on zebrafish early embryonic development.

## INTRODUCTION

Oxygen (O_2_) is a fundamental prerequisite to maintain cellular homeostasis for all aerobic organisms [1]. Consequently, hypoxia, a unique physiological stimulus, is implicated in a wide range of biological and cellular processes, such as cell survival [2], cell proliferation and invasion [3], angiogenesis [4], and neuronal development [5]. The cellular adaptive responses to hypoxic stress are mainly mediated by the hypoxia-inducible factors (HIFs) which are heterodimeric complexes consisting of an oxygen-labile α subunit (HIF-1α, HIF-2α and HIF-3α) and a constitutively expressed β-subunit (HIF-1β/ARNT) [6, 7]. HIF-1α has been identified as the best characterized oxygen sensor and regulator of the hypoxic-adaptive responses that preserve cell viability [8, 9]. Under normoxic conditions, HIF-1α is hydroxylated by proline-hydroxylase-2 (PHD2) whose activity is oxygen-dependent [10, 11], subsequently recognized by the von-Hippel-Lindau (pVHL) tumor suppressor protein and subjected to proteasomal degradation [12, 13]. Accordingly, under hypoxic conditions, owing to the suppression of hydroxylation, HIF-1α accumulates and dimerizes with HIF-1β. This heterodimer binds to hypoxia response elements (HREs) and transcriptionally activate a large group of hypoxia-sensitive genes [14, 15], which are involved in cell differentiation and apoptosis [16], migration and invasion [17], erythropoiesis [18] and angiogenesis [19]. In addition, hypoxia and HIFs are also known to play important roles in tissue formation and embryo morphogenesis [20–23].

It is revealed that a specific subset of microRNAs (miRNAs) is induced by HIF-1 under hypoxic conditions and, in turn, controls the expression of HIF-1, which fine-tunes cellular adaptations to hypoxia [24]. MiRNAs are endogenous noncoding small RNAs (∼22 nucleotides in length) which regulate gene expression at the post-transcriptional level [25, 26]. Recent works indicate that hypoxic stress regulates the expression of a number of miRNAs, termed hypoxamirs, either in a HIF-dependent or HIF-independent manner [27, 28]. A number of these hypoxia-regulated miRNAs are demonstrated to contribute to cellular responses to hypoxia by modulating critical downstream targets, including let-7 [29], miR-429 [30], miR-195 [31], miR-210 [32, 33], miR-322 [34], miR-200a [35], miR-199a [36, 37] and miR-150 [38].

The miR-125 family, a highly conserved miRNA family throughout evolution, has been demonstrated to be implicated in a variety of physiological processes, including cell proliferation and apoptosis [39, 40], cell metastasis [41] and immune response [42]. Recent studies have exhibited the dual function of miR-125 family as tumor suppressor and promotor, and the aberrant expression of miR-125 family is tightly related to tumorigenesis [43, 44]. Furthermore, miR-125a is demonstrated to be hypoxia-induced in human cancer [7]. Interestingly, miR-125c is a unique homolog which is absent in mammals [43, 44]. And our data showed that miR-125c was up-regulated by hypoxia both in zebrafish embryos and ZF4 cells. In the present study, to exhibit the regulatory and biological roles of miR-125c in hypoxia adaptation, we detected the transcriptional regulation of Hif-1α on miR-125c and identified *cdc25a* as a novel downstream target. In addition, we found that miR-125c suppresses cell proliferation through cell cycle arresting, and induces apoptosis, providing a Hif-1α/miR-125c/*cdc25a* signaling which may function in cellular adaptations to hypoxia. Additionally, for the first time, we characterized the function of miR-125c in zebrafish normal embryogenesis.

## RESULTS

### MiR-125c is transcriptionally induced by Hif-1α

**Figure 1:**
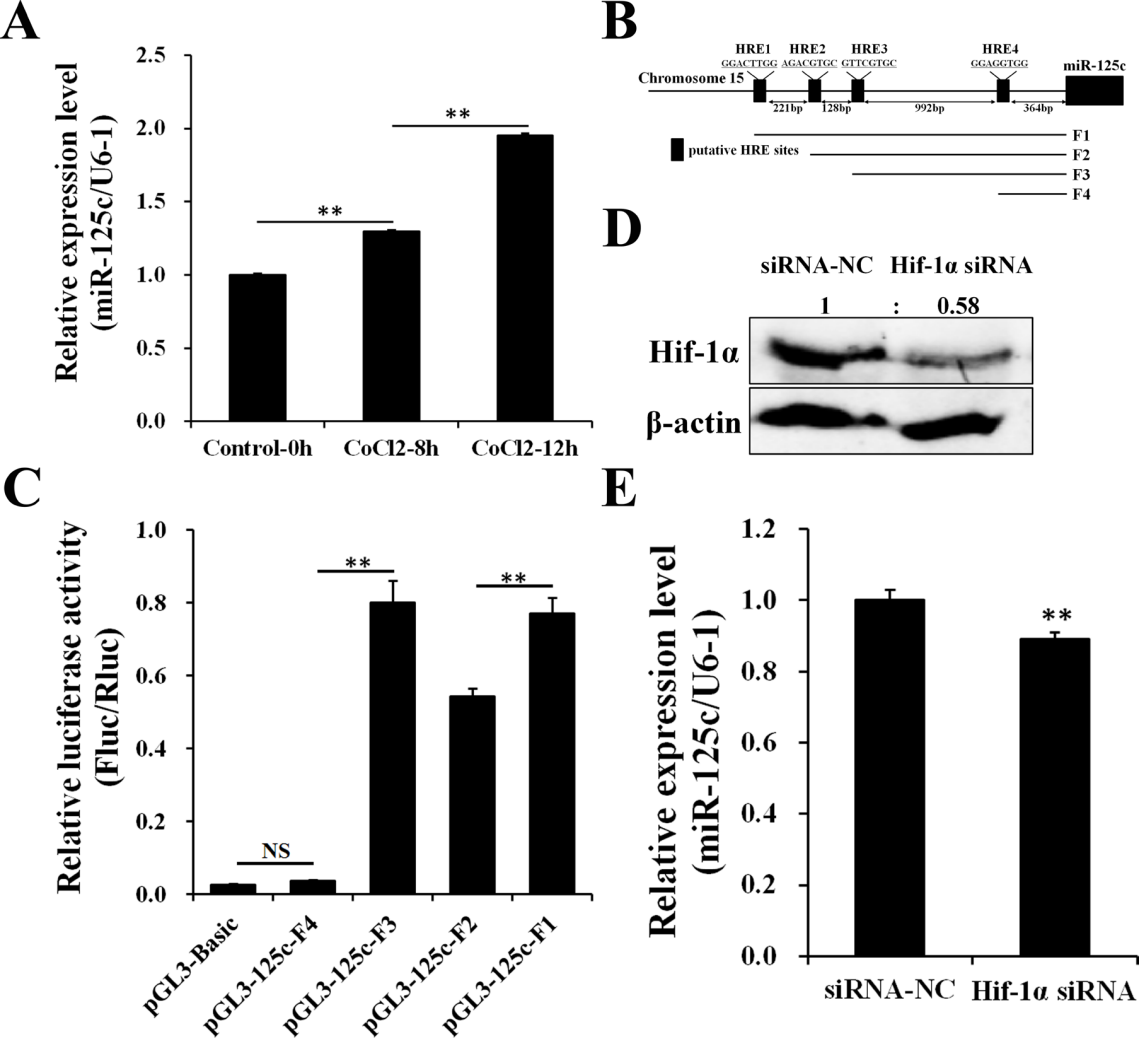
Hif-1α contributes to the transcription of miR-125c (**A**) Expression of miR-125c in ZF4 cells treated with 100 µM CoCl_2_ for 0 h, 8 h and 12 h before being collected. U6-1 is used as the endogenous control. Values represent means ± S.D. (*n* = 3, **P < 0.01). (**B**) A schematic depiction of the miR-125c promoter region. Location and sequence information of four putative HREs (HRE1, HRE2, HRE3 and HRE4) are indicated. A series of promoter fragments (F1, F2, F3 and F4) carrying different HRE motifs were amplified. (**C**) The luciferase reporters harboring different HREs was cotransfected with the pRL-TK *Renilla* luciferase reporter (internal control) and pCMV-Myc-Hif-1α into HeLa cells. Luciferase activities were detected after stimulating with 100 μM CoCl_2_ for 4 h, and *Firefly* luciferase expression was normalized to *Renilla* luciferase. Results are presented as mean ± S.E. (*n* = 3, **P < 0.01, NS, not significant). (**D**) Protein detection of Hif-1α in ZF4 cells transfected with Hif-1α siRNA (siRNA-NC, negative control siRNA) and treated with 100 μM CoCl_2_ for 12 h before harvest (hypoxia-simulating). β-actin is used to normalize protein levels. Numbers indicate quantification of the Hif-1α band densities relative to β-actin. (**E**) Expression of miR-125c in ZF4 cells transfected with Hif-1α siRNA and treated with 100 μM CoCl_2_ for 12 h before harvest. U6-1 is used as the endogenous control. Values represent means ± S.D. (*n* = 3, **P < 0.01).

Our previous date showed that miR-125c was up-regulated by acute hypoxia in zebrafish embryos exposed to the low oxygen level of 1.0 mg/L by filling nitrogen continually, from 34 hpf to 36 hpf (Supplementary Figure 1). In addition, miR-125c was significantly induced in a time-dependent manner in response to hypoxia-simulating treatment with CoCl_2_ in ZF4 cells (Figure 1A), along with the accumulation of Hif-1α [45]. Firstly, we identified four putative HRE motifs on the promoter region of miR-125c from 2 kb upstream of the transcriptional start site (TSS) (Figure 1B). To investigate whether these HREs could respond to Hif-1α and contributes to the up-regulation of miR-125c under hypoxic condition, luciferase reporter assay was performed. As shown in Figure 1C, the HRE1 and HRE3 can be effectively targeted by Hif-1α, resulting in the significant increase of corresponding reporter activity. On the other hand, HRE2 and HRE4 do not contribute to the transcription of miR-125c. To further demonstrate the transcriptional activation of miR-125c by Hif-1α, knockdown of Hif-1α was performed by siRNA transfection in ZF4 cells under hypoxic condition (CoCl_2_ treatment for 12 h). As shown in Figure 1D, accumulation of endogenous Hif-1α was effectively inhibited. Accordingly, the expression level of miR-125c was slightly but significantly decreased (Figure 1E).

### MiR-125c directly targets *cdc25a* which responds to cellular hypoxia

**Figure 2:**
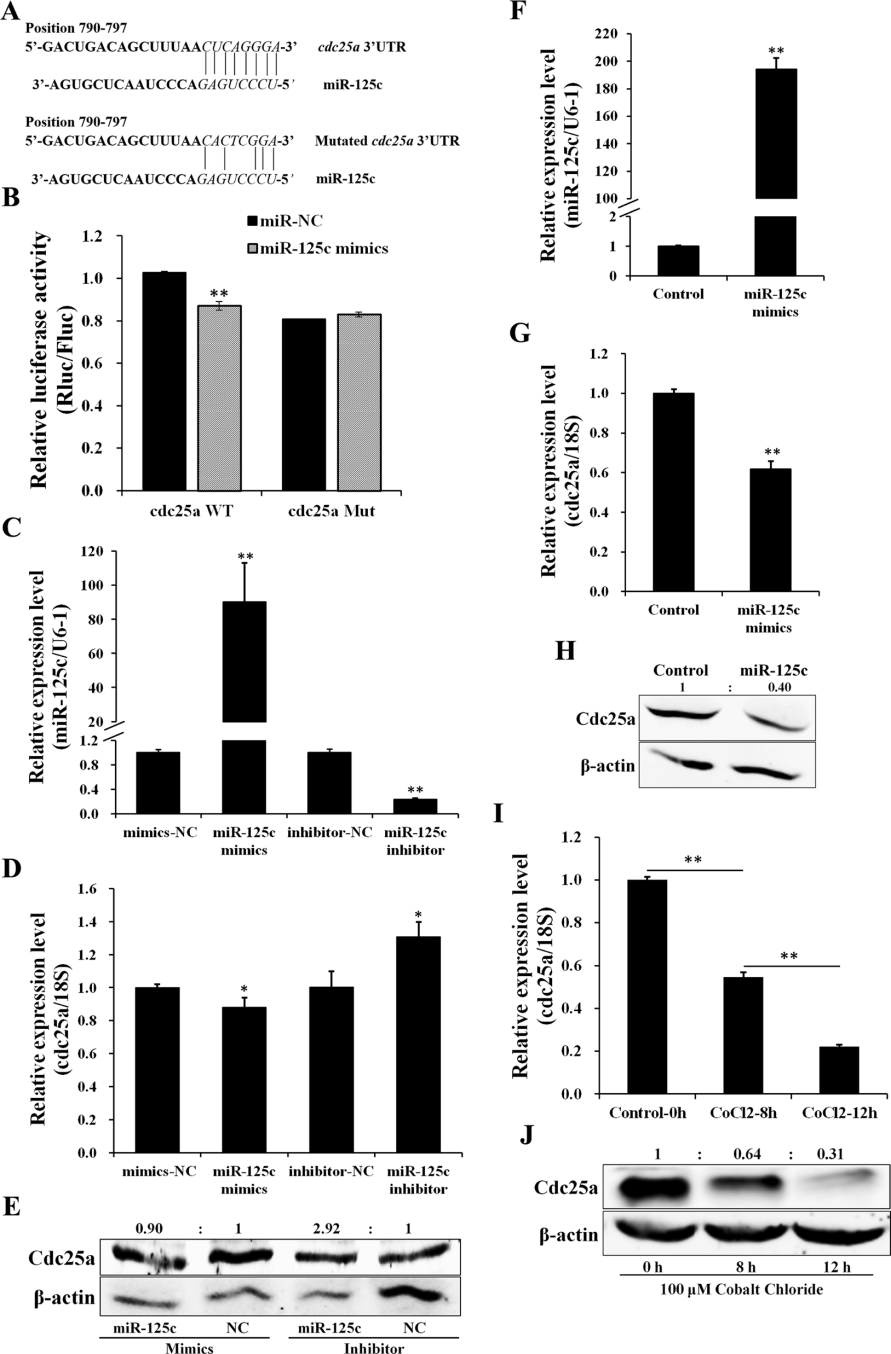
miR-125c directly targets cdc25a which responds to cellular hypoxia (**A**) Schematic illustration of *cdc25a* 3′UTR fragment harboring a miR-125c binding site (highlighted in italics). The sequence information of the *cdc25a* 3′UTR in wild type (WT) and its mutant (Mut) with disrupted base pairing are indicated. (**B**) Dual-luciferase reporter assay for validation of miR-125c binding site on the 3′UTR of cdc25a. HeLa cells were cotransfected with *cdc25a* 3′UTR dual-luciferase constructs (WT or Mut) plus miR-125c mimics. *Renilla* luciferase activities were detected and normalized to *Firefly* luciferase (internal control). Results represent means ± S.E. (*n* = 3, **P < 0.01). (**C**) Transfection efficiency of miR-125c mimics and inhibitor in ZF4 cells was detected by qRT-PCR. All data are means ± S.D. (*n* = 3, **P < 0.01). (**D**) The *cdc25a* expression in ZF4 cells transfected with miR-125c mimics or inhibitor was detected by qRT-PCR. 18s rRNA is used as the endogenous control. Values represent means ±S.D. (*n* = 3, *P < 0.05). (**E**) Expression analysis of Cdc25a protein by western blot in corresponding ZF4 cell samples. β-actin is used to normalize protein levels. Numbers indicate quantification of the Cdc25a band densities relative to β-actin. (**F**) Injection efficiency of miR-125c mimics in zebrafish embryos was detected by qRT-PCR. U6-1 is used as the endogenous control. Values represent means ±S.D. (*n* = 3, **P < 0.01). (**G**) The regulation of *cdc25a* expression by miR-125c in zebrafish embryos based on qRT-PCR. 18 s rRNA is used as the endogenous control. Values represent means ±S.D. (*n* = 3, **P < 0.01). (**H**) The regulation of Cdc25a protein expression by miR-125c in zebrafish embryos based on western blot analysis. β-actin is used to normalize protein levels. Numbers indicate quantification of the Cdc25a band densities relative to β-actin. (**I**) Expression of *cdc25a* in ZF4 cells treated with 100 µM CoCl_2_ for 0 h, 8 h and 12 h before being collected. 18 s rRNA is used as the endogenous control. Values represent means ±S.D. (*n* = 3, **P < 0.01). (**J**) Expression analysis of Cdc25a protein by western blot in corresponding ZF4 cell samples. β-actin is used to normalize protein levels. Numbers indicate quantification of the Cdc25a band densities relative to β-actin.

Based on TargetScan bioinformatic algorithm, *cdc25a* was identified as a potential target of miR-125c, with a complementary binding site on the 3′UTR (Figure 2A). To validate the direct targeting of *cdc25a* by miR-125c, the dual-luciferase assay was carried out, showing that miR-125c overexpression was associated with the reduction of the luciferase activity. The specificity of this inhibition was demonstrated by the finding that the activity of a mutant *cdc25a* 3′UTR construct was not affected (Figure 2B). To determine the inhibition of *cdc25a* by miR-125c, overexpression and knockdown were conducted in ZF4 cells. The transfection efficiency was determined by qRT-PCR (Figure 2C). The *cdc25a* mRNA and protein levels were clearly decreased by miR-125c mimics, but increased by miR-125c inhibitor (Figure 2D, 2E). We further confirmed the negative regulation of *cdc25a* by miR-125c in zebrafish embryos, and the result showed that ectopic expression of miR-125c resulted in a dramatic reduction of both *cdc25a* mRNA and protein levels (Figure 2F–2H).

Furthermore, we found that, contrary to miR-125c, the endogenous *cdc25a* mRNA and protein levels were significantly down-regulated in a time-dependent manner in response to hypoxia-simulating treatment with CoCl_2_ in ZF4 cells (Figure 2I, 2J).

### MiR-125c represses cell proliferation through cell-cycle arresting and induces apoptotic responses

**Figure 3:**
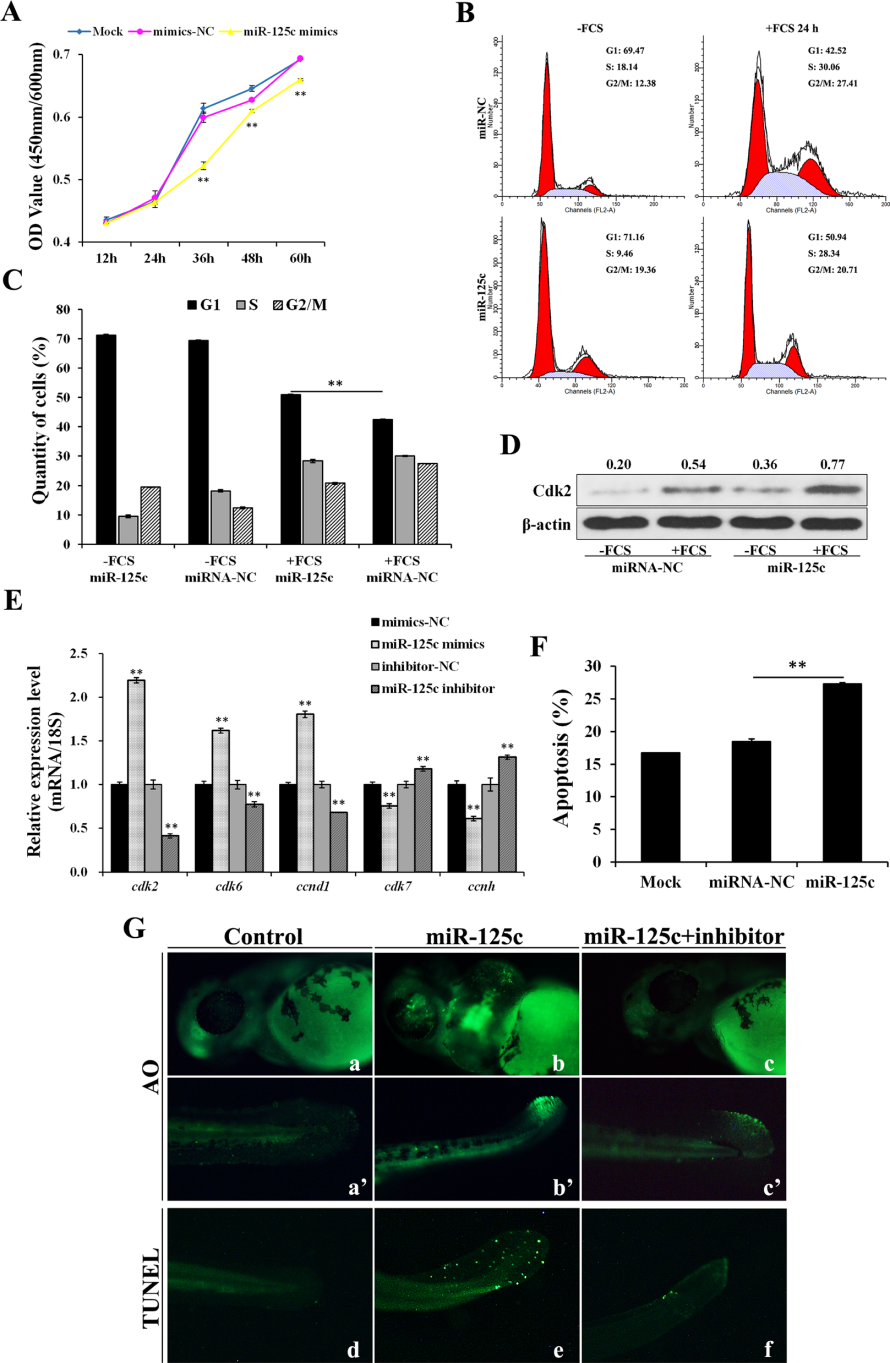
miR-125c represses cell proliferation through cell-cycle arresting at G1 phase and induces cell apoptosis (**A**) ZF4 cells were transfected with miR-125c mimics (mimics-NC, negative control), and cell proliferation index was continuously detected using CCK-8 assays kit. Mock represents transfection control. All of the values represent the means ± S.D. (*n* = 3, **P < 0.01). (**B**) After 24 h transfection with miR-125c mimics (miR-NC, negative control), ZF4 cells were synchronized in G0/G1 by FCS starvation for 35 h (-FCS) and launched into cell cycle process by FCS addition for another 24 h (+FCS). The cell cycle analysis was performed in triplicate by flow cytometry. The data correspond to the mean of three independent experiments. (**C**) Percentage of ZF4 cells in G1, S, and G2/M phase were determined. The results are presented as means ± S.E. (*n* = 3, **P < 0.01). (**D**) Expression analysis of Cdk2 protein by Western blot in corresponding ZF4 cell samples. β-actin is used to normalize protein levels. Numbers indicate quantification of the Cdk2 band densities relative to β-actin. (**E**) Expression analysis of cell cycle genes *cdk2*, *cdk6*, *ccnd1*, *cdk7* and *ccnh* in ZF4 cells transfected with miR-125c mimics (mimics-NC, negative control for mimics) or inhibitor (inhibitor-NC, negative control for inhibitor). 18s rRNA is used as the endogenous control. Values represent means ± S.D. (*n* = 3, **P < 0.01). (**F**) Percentage of apoptosis in ZF4 cells transfected with miR-125c mimics (miR-NC, negative control) was determind by Annexin V-FITC/PI staining and flow cytometry. Mock represents transfection control. The results are represented as means ± S.E. (n = 3, **P < 0.01). (**G**) Detection of cell death and apoptosis phenotypes in miR-125c injected zebrafish embryos at 48 hpf by AO staining (a-c and a’-c’) and TUNEL assays (d-f), respectively. Injection of miR-125c mimics together with inhibitor was performed to determine whether knockdown of miR-125c can rescue the apoptosis phenotype.

Cell Counting Kit-8 (CCK8) assays were performed to assess the effect of miR-125c on cell proliferation. The result revealed that, compared with negative control, miR-125c significantly (*P* < 0.01) suppressed the proliferation of ZF4 cells from 36 h to 60 h (Figure 3A). Since several evidences have shown that hypoxia inhibits cell cycle progression by controlling the expression of critical cell cycle genes including *CDC25A*, we further investigate whether miR-125c contributes to the hypoxic blockade of cell cycle progression. The flow cytometry assay revealed that ZF4 cells were effectively synchronized at G0/G1 phase (∼70%) by serum starvation, and released from quiescence by fetal calf serum (FCS) addition. As shown in Figure 3B and 3C, miR-125c significantly blocked the cell cycle progression at G1 phase. Meanwhile, we observed a strong accumulation of the canonical G1/S transition regulator Cdk2 by miR-125c overexpression (Figure 3D), leading us to explore the function of miR-125c in cell cycle regulation by assessing the expression of various cell cycle genes, including the G1 phase regulatory genes *cdk2, cdk6*, *ccnd1* and S phase regulatory genes *cdk7*, *ccnh*. It was revealed that the *cdk2*, *cdk6*, *ccnd1* mRNA levels were significantly increased by miR-125c mimics but down-regulated by the miR-125c inhibitor. On the contrary, the *cdk7* and *ccnh* mRNA levels were strongly decreased by miR-125c mimics but increased by the miR-125c inhibitor (Figure 3E).

Additionally, the effect of miR-125c on cell apoptosis was assessed and the results showed that miR-125c overexpression in ZF4 cells resulted in a significant increase of the apoptosis level (Figure 3F). Accordingly, we evaluated the cellular uptake of acridine orange, a general indicator of cell death, in the living zebrafish embryos at 48 hpf. Compared with the control (Figure 3G-a, a’), miR-125c overexpression gave rise to a significant increase of apoptotic cells in brain, eyes and tail (Figure 3G-b, b’) that could be partially rescued by the miR-125c inhibitor (Figure 3G-c, c’). In addition, cell apoptosis in tail was further confirmed by TUNEL assay, which could also be rescued by the miR-125c inhibitor (Figure 3G-d, e, f).

### MiR-125c is essential for normal embryogenesis of zebrafish

**Figure 4:**
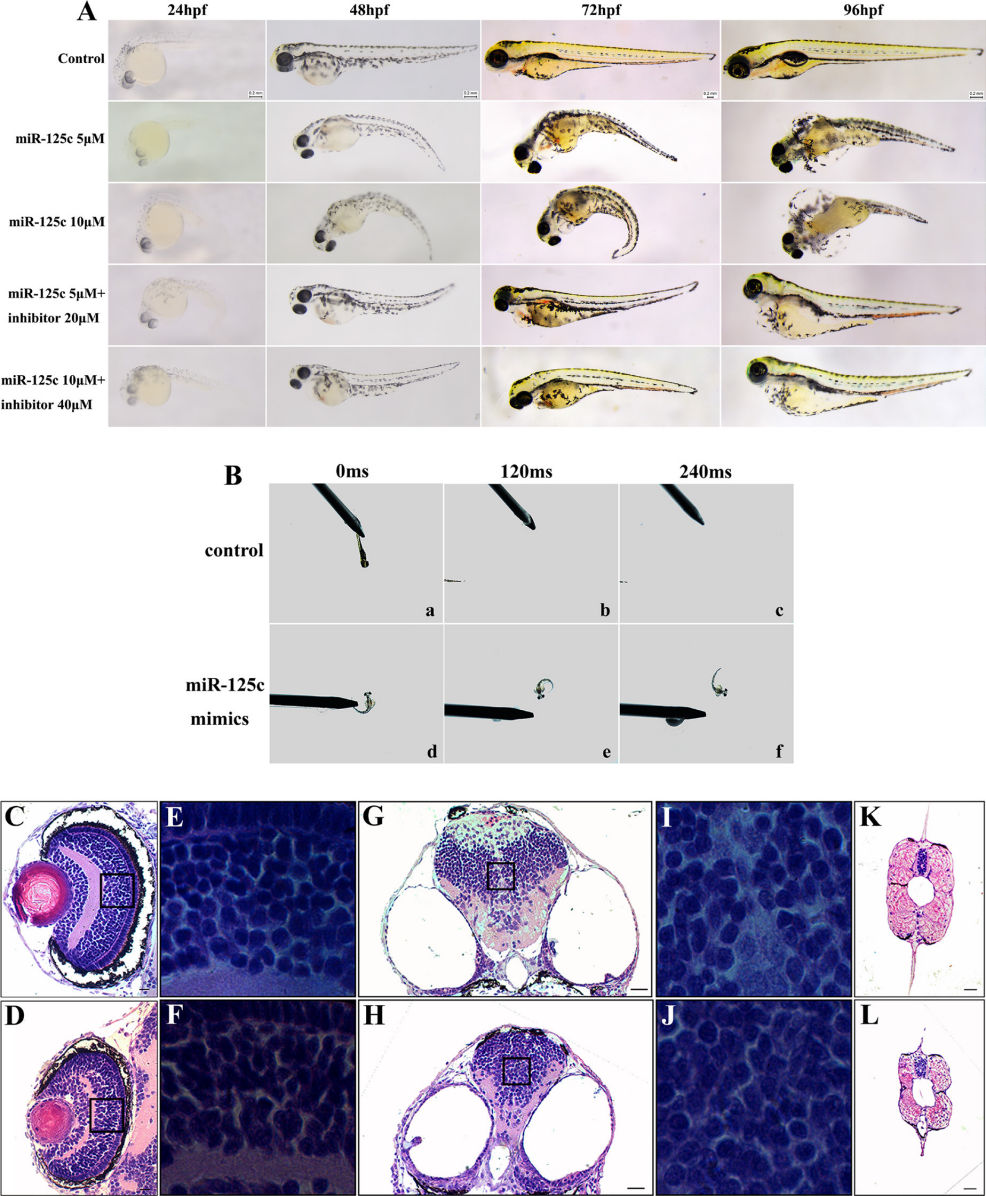
Overexpression of miR-125c results in severe abnormalities and reduction of motility during embryonic development (**A**) miR-125c injection induces distinct embryonic malformation in a dose dependent manner during early development (24–96 hpf). Injection of miR-125c mimics together with inhibitor was performed to determine whether knockdown of miR-125c can rescue the morphological defects. Scale bars, 0.2 mm. (**B**) miR-125c injected embryos exhibit diminished swimming ability in response to mechanosensory stimulation at 3 dpf. a-c) Touching the tail provokes the wild-type embryos to swim away rapidly (within 120- 240 ms) (*n* = 5). d-f) But, miR-125c injected embryos swim slowly in circular motions (*n* = 5). (**C**, **D**) Histological analysis of the eye in miR-125c injected embryos at 4 dpf reveals smaller lens and thinner inner plexiform layer (IPL). Scale bars, 20 µm. (**E**, **F**) Magnified views shows clumped and disorganized cells in the inner nuclear layers (INL) in miR-125c injected embryos. (**G**, **H)** Smaller brain is observed in miR-125c injected embryos at 4 dpf. Scale bars, 40 µm. (**I**, **J**) Magnified views reveals tightly clumped cells with reduced extracellular space in the miR-125c injected embryos. (**K**, **L**) miR-125c injected embryos at 4 dpf exhibit smaller myotome and degenerated muscle fibers. Scale bars, 40 µm.

Our results suggested that miR-125c represses ZF4 cell proliferation and induces apoptosis in zebrafish embryos, making it necessary to further determine the potential function of miR-125c in embryogenesis. Therefore, we observed morphological changes induced by microinjection of miR-125c mimics. From 24 hpf, compared to wild-type embryos, miR-125c overexpressed embryos showed pericardial edema, severely impaired head with concomitant reduction of eyes, and abnormally curved tail (Figure 4A). The malformation was observed in a dose-dependent manner and can be partially rescued by miR-125c inhibitor (Figure 4A). The pericardia edema phenotype was first recognized at around 72 hpf, with a tiny heart and bradycardia compared with wild-type embryos. The miR-125c overexpressed embryos also exhibited slower heart rate with the average of 138 beats/min (*n* = 145), compared with the wild-type (173 beats/min, *n* = 126).

Furthermore, we assessed the motor ability of zebrafish embryos by touch evoked response assay. We observed that miR-125c overexpressed embryos showed diminished swimming ability in response to mechanosensory stimulation at 3 dpf (Supplementary Videos 1 and 2). Touching the tail provoked the wild-type embryos to swim out of the field of view rapidly (witin 120–240 ms), but miR-125c injected embryos swam slowly in circular motions (Figure 4B, Supplementary Videos 1 and 2).

Finally, to evaluate the defects in miR-125c overexpressed embryos further, histological examination was conducted. It was revealed that the lens appeared smaller and the inner plexiform layer (IPL) in eyes became thinner in these embryos (Figure 4C, 4D). Meanwhile, the magnified views showed clumped and irregularly arranged cells in inner nuclear layers (INL) (Figure 4E, 4F). Besides, miR-125c overexpression resulted in a reduction of brain size with tightly clumped cells and decreased extracellular space (Figure 4G–4J). Additionally, in miR-125c injected embryos, smaller myotome and degenerated muscle was seen (Figure 4K, 4L), which may be responsible of the reduced motility. These results indicated that miR-125c is essential for normal embryogenesis in zebrafish.

## DISCUSSION

Hypoxia is one of the most important microenvironmental factors and occurs in various physiological and pathological processes, especially in tumor [46]. In order to cope with limitation in availability of oxygen, organisms undergo a variety of cellular responses [2] and a highly coordinated regulation of hypoxia-responsive gene expression [47]. Recently, a specific set of hypoxia-regulated miRNAs, termed hypoxamirs, have been identified to regulate cellular adaptation to hypoxia via controlling target gene expression at the post-transcriptional level [25–28]. In the present study, we have identified miR-125c as a hypoxia-induced miRNA in zebrafish and revealed its significant roles in regulating cell survival and embryogenesis.

Hypoxia is a crucial tumor microenvironment that evokes highly coordinated cellular responses. Recently, miR-125 has been confirmed to be hypoxia-induced in human cancer cells [7], also it was demonstrated to function in the transgenerational effect of hypoxia in medaka testis [48]. But miR-125c is a unique homolog which is absent in mammals and little is known about its regulatory and physiologic functions in hypoxia adaptation. HIF-1α is a master transcriptional regulator that controls the cellular responses to hypoxia by targeting a number of downstream genes [8, 9]. Recently, emerging evidence revealed that numerous hypoxamirs are hypoxia-induced through the HIF-1α-dependent mechanism, among which miR-210 is the most canonical and influential one [27, 28, 49]. Our previous date showed that miR-125c was up-regulated by hypoxia in zebrafish embryos (Supplementary Figure 1). In addition, miR-125c was significantly induced in a time-dependent manner in response to hypoxia-stimulating with CoCl_2_ in ZF4 cells. To determine whether Hif-1α contributes to the hypoxic induction of miR-125c, we performed a luciferase assay and confirmed the transcriptional activation of miR-125c by Hif-1α. Additionally, the upregulation of miR-125c expression by hypoxia-stimulating in ZF4 cells was abolished by knockdown of Hif-1α, further demonstrating that the hypoxia-induced expression of miR-125c in zebrafish is mediated via the Hif-1α pathway.

It has been well known that miRNAs can regulate large number of target genes at the post-transcriptional level, consequently, inducing diverse cellular processes, such as cell proliferation, differentiation, and apoptosis [27, 50, 51]. MiR-125 family is highly conserved throughout evolution, and it was demonstrated to regulate tumorigenesis and tumor development by targeting important genes including transcription factors like CBFB and TGF-β [52, 53], anti-apoptotic genes like BCL2, BCL2L12 and Mcl-1 [54], pro-apoptotic protein Bak1 [55], and tumor suppressing protein p53 [55, 56]. Since miR-125c is a unique homolog which is not reported in mammals, we characterized the sequence conservation of the miR-125 family in zebrafish, medaka, human and mouse by multiple sequence alignment which revealed that the seed sequences of miR-125a/b/c were fully conserved (Supplementary Figure 2). Recent study indicated that *CDC25A*, a critical cell cycle gene, is responsive to cellular hypoxia in human cancer, and HIF-1α-Myc pathway is also involved in the hypoxic downregulation of *CDC25A* [57]. Our previous date showed that *cdc25a* was downregulated by hypoxia in zebrafish embryos (Supplementary Figure 1). In addition, contrary to miR-125c, the endogenous *cdc25a* mRNA and protein levels were significantly suppressed in a time-dependent manner in response to hypoxia-simulating with CoCl_2_ in ZF4 cells. Interestingly, we identified a putative binding site of miR-125 on zebrafish *cdc25a* 3′UTR by TargetScan, which is absent on the *CDC25A* 3′UTR of both human and mouse. These findings prompted interest in understanding whether zebrafish miR-125c contribute to the hypoxic inhibition of *cdc25a*. Our results demonstrated that miR-125c can repress endogenous Cdc25a mRNA and protein expression by binding the 3′UTR both in ZF4 cells and zebrafish embryos, which confirmed zebrafish *cdc25a* as a novel downstream target of miR-125c. Taken together, our findings suggested a novel Hif-1α/miR-125c signaling for hypoxic repression of *cdc25a*.

CDC25A, a dual-specificity phosphatase, is one of the most crucial cell cycle regulators, which is involved in tumorigenesis, and plays important roles in DNA replication, cell proliferation and apoptosis [58]. Cdc25A plays a extensive role in assisting both G1/S and G2/M progression by dephosphorylating CDK4, CDK6 as well as CDK2 and CDK1 [59, 60]. Evidence has shown that hypoxia can block cell cycle progression at the G1/S transition by controlling the expression of critical cell cycle genes including *CDC25A* [57]. Our results showed that miR-125c overexpression significantly suppressed cell proliferation of ZF4 cells, induced G1/S cell cycle arrest and promoted cell apoptosis. Additionally, the mRNA levels of S phase regulatory genes *cdk7* and *ccnh* were also downregulated by miR-125c. However, it was observed that overexpression of miR-125c upregulated the expression of three G1 phase regulatory genes *cdk2*, *cdk6* and *ccnd1*, which may be result from its inhibition of another potential target p53. It was reported that miR-125 family has a dual function in suppression and promotion of cancer cells [44]. Evidences demonstrated that, in some cases, miR-125 could directly downregulate p53 and further influence the downstream protein p21 which is a CDK inhibitor [61, 62]. Based on TargetScan bioinformatic algorithm, zebrafish p53 was also identified as a potential target of miR-125 with a putative binding site on the 3′UTR, which is not verified in the current study but provides a new direction for the follow-up study. Overall, our findings suggested that miR-125c may regulates cell survival at least in part through inhibition of *cdc25a*, but further studies are needed to identify additional targets of miR-125c to fully understand the complex regulatory mechanisms in cellular adaptation to hypoxia.

Apart from inducing cell cycle arrest, miR-125c overexpression in zebrafish embryos resulted in cell apoptosis in brain, eyes and tail, implying an essential role for miR-125c in normal embryogenesis. Recently, miRNAs have been confirmed to regulate zebrafish morphogenesis and differentiation [63], somitogenesis [64], heart [65] and neural development [66]. Both miR-125a and miR-125b play excellent roles in the zebrafish brain development [67], meanwhile miR-125b also regulates the zebrafish eye development [67]. Here, for the first time, we observed that miR-125c-overexpressed embryos exhibit defective phenotypes, including smaller eyes, impaired brain, curved tail and trunk. Moreover, injection of miR-125c caused bradycardia accompanied with pericardial edema, suggesting its effect on cardiac function. It has been revealed that deficiency of skeletal muscle fibers in zebrafish embryos at early stages will lead to reduction of motility [68]. In this work, overexpression of miR-125c resulted in degenerating muscle fibers and embryo immotility or unusual circular motions. Additionally, evidences revealed that Cdc25a activity needs to be carefully regulated to preserve normal occurrence of particular morphogenetic events, and restricted expression of Cdc25a is required for somitogenesis and muscle fiber formation [69]. It is possible that miR-125c has a significant influence on the skeletal muscle differentiation through regulation of Cdc25a, but further studies are needed to support this hypothesis. Taken together, these results suggested an essential role for miR-125c in zebrafish normal embryogenesis.

It is reasonable that the responses to hypoxia ultimately contribute to embryonic morphogenesis through effects on cell proliferation, cell differentiation and cell behavior. The understanding of the role of HIF in the cellular adaptation to hypoxia during embryonic development is still unfolding. A number of evidences have demonstrated its effect on tissue formation and many developmental systems, such as hematopoiesis [70], angiogenesis [71], hepatogenesis [72], bone formation [73] and heart development [20]. In vertebrate embryos, regulation of the entry into mitosis is essential for normal morphogenesis, and *cdc25* is a key controller of mitotic entry. It has been demonstrated that *cdc25a* can accelerate the entry of post-blastoderm cells into mitosis, suggesting that levels of *cdc25a* are rate limiting for cell cycle progression during zebrafish gastrulation [74]. In addition, restricted expression of *cdc25a* in the tailbud during the somite-forming stage is essential for muscle cell differentiation and morphogenesis of the zebrafish posterior body [69]. In the present study, we have suggested that miR-125c has an indispensable function during zebrafish muscle differentiation partially through regulation of Cdc25a. But it is unprecise to deduce that all the severe malformations are solely resulted from *cdc25a* downregulation, and further studies are needed to reveal the mechanism through which miR-125c affects embryogenesis.

In conclusion, we identified zebrafish miR-125c as hypoxia-induced via the Hif-1α-mediated manner. Through gain-of-function and rescue approaches, we provide a novel Hif-1α/miR-125c/*cdc25a* signaling that plays essential roles in cellular adaptations to hypoxia, and suggest a novel function of miR-125c in zebrafish normal embryogenesis.

## MATERIALS AND METHODS

### Fish husbandry

Adult AB strain zebrafish (*Danio rerio*) were raised and maintained on a 14/10 h light–dark schedule in the recirculating water system (28 ± 1°C). Embryos at different development stages were collected as described previously [75].

### Plasmid construction

The 2kb region upstream from the TSS of miR-125c was obtained using the UCSC Genome Browse. Then the JASPAR CORE Vertebrate database was used to search for potential HREs on the promoter region [76]. The promoter fragments of miR-125c containing different indicated HREs, including F1 (HRE1-4), F2 (HER2-4), two F3 (HRE3, 4) and F4 (HRE4), were amplified and directly inserted into the pGL3-Basic vector (Promega, USA). The recombinant expression vector pCMV-Myc-Hif-1α, expressing recombinant zebrafish Myc-tagged-Hif-1α protein, was constructed in our previous study [45].

The wild-type or mutant 3′UTR fragment of *cdc25a* (cell division cycle 25a, GenBank: NM_001115095.2) containing the putative miR-125c binding site were amplified and subcloned into the psiCHECK-2 dual-luciferase reporter vector (Promega). All primers used for plasmid construction were listed in Table 1.

**Table 1:** Primers used in qRT-PCR and plasmid construction

Primer name	Primer sequence (5′–3′)
ZB-qRT-*cdc25a*-F	CCTCCATTACCCCGAACTCT
ZB-qRT-*cdc25a*-R	GTGCGGCTCTTCAGACGAAA
ZB-qRT-*cdk2*-F	TCGGAGAGGGAACATACG
ZB-qRT-*cdk2*-R	TTCACGAGTGGCAAGGAT
ZB-qRT-*cdk6*-F	AGTGCCACCTGAAACCAT
ZB-qRT-*cdk6*-R	CACGGAAGTAAGAGCCATC
ZB-qRT-*ccnd1*-F	CCTGTTGAATGACCGAGTT
ZB-qRT-*ccnd1*-R	CTGGTTTTTTTGGTGGGC
ZB-qRT-*cdk7*-F	GGAAGCCCAAACAGAGT
ZB-qRT-*cdk7*-R	TGTCCACACCTACACCATA
ZB-qRT-*ccnh*-F	TAAGCCTGTGATGCCTAA
ZB-qRT-*ccnh*-R	ATACGGATTGTGAACGACC
ZB-qRT-18s rRNA-F	CGGAGGTTCGAAGACGATCA
ZB-qRT-18s rRNA-R	GGGTCGGCATCGTTTACG
qRT-miR-125c-F	GGCTGCTCCCTGAGACCCTAACT
qRT-miR-125c-R qRT-miR-125c-Stem loop	TCAACTGGTGTCGTGGAGTCGGC CTCAACTGGTGTCGTGGAGTCGGCAATTCA GTTGAGTCACGAGT
qRT-U6-F	TGCTCGCTACGGTGGCACA
qRT-U6-R	AAAACAGCAATATGGAGCGC
ZB-*cdc25a*-3′UTR-F	CTCGAGGTTGCTGAATGTAACTAATCTG
ZB-*cdc25a*-3′UTR-Rm	CAGTCCGAGTGTTAAAGCTGTCAGT
ZB-*cdc25a*-3′UTR-Fm	GACTGACAGCTTTAACACTCGGACTG
ZB-*cdc25a*-3′UTR-R	CAGTCCGAGTGTTAAAGCTGTCAGT
ZB-miR-125c-pro-F1	CGCGACGCGTGCTTTAAAAGTCCCCCGGC
ZB-miR-125c-pro-F2	CGCGACGCGTATCGTTACACGCACCCTCT
ZB-miR-125c-pro-F3	CGCGACGCGTCAGCACTGGTGTAAATGAT
ZB-miR-125c-pro-F4	CGCGACGCGTCCGATGGGGATTTTTCTGT
ZB-miR-125c-pro-R	CCCCAAGCTTATCGCTTCTCTTCCGTCAC

### Cell culture and hypoxia-mimicking treatment

HeLa and ZF4 cells (Cell Collection Center for Freshwater Organisms, Huazhong Agricultural University) were maintained at 37°C and 28°C in a 5% CO_2_ atmosphere in DMEM and DMEM/F12 1:1 medium (Hyclone, USA) supplemented with 10% fetal bovine serum and 1% penicillin-streptomycin, respectively.

To induce endogenous Hif-1α protein by mimicking hypoxic condition, ZF4 cells cultured in 6-well plates were treated with 100 µM [45] cobalt chloride (CoCl_2_, Sigma, USA) for 0 h (control), 8 h and 12 h in turns before being collected for isolation of total RNA (including miRNA) and protein at 24 and 48 h after the cultivation, respectively. For HRE activity assay, HeLa cells were stimulated with 100 μM CoCl_2_ for 4 h to induce recombinant zebrafish Myc-tagged-Hif-1α protein as previous described [45].

### Cell transfection and luciferase reporter assays

For miR-125c overexpression and knockdown in ZF4 cells, miR-125c mimics (80 nM), mimics-NC (80 nM), miR-125c inhibitor (120 nM), and inhibitor-NC (80 nM) (GenePharma, China) were transfected separately. Total RNA (including miRNA) and protein were isolated at 24 h and 48 h post transfection, respectively. For Hif-1α knockdown, ZF4 cells were transfected with 120 nM Hif-1α siRNA (Sense: 5′- GGTAGACGCTTAAGGTAATdTdT-3′) (GenePharma), then treated with 100 µM CoCl_2_ [45] for 12 h before being collected for total RNA (including miRNA) and protein isolation.

For HRE activity verification, HeLa cells cultured in 24-well plates were cotransfected with pCMV-Myc-Hif-1*α*, indicated recombinant HRE-luciferase repoter plasmids pGL3b-F1/F2/F3/F4, and the pRL-TK *Renilla* luciferase reporter (internal control) (Promega). The cells were then stimulated with 100 µM CoCl_2_ for 4 h before the luciferase assay at 24 h post transfection [45]. Relative luciferase activities were determined by normalizing *Firefly* activity to *Renilla* activity. For the target detection of miR-125c, miRNA mimics or miRNA-NC (negative control) and *cdc25a* 3′UTR dual-luciferase constructs (WT or Mut) were cotransfected into HeLa cells. The luciferase activity was measured at 24 h post transfection using the Dual-Luciferase Reporter Assay System (Promega), and *Renilla* luciferase activity was normalized to *F*irefly luciferase. All transfections were performed using Lipofectamine 2000 (Invitrogen, USA).

### Cell proliferation, cell cycle and cell apoptosis assays

ZF4 cells were seed in a flat-bottom 96-well plate and transfected separately with 50 nM miR-125c mimics and mimics-NC. Subsequently, cell proliferation rates were measured at 12, 24, 36, 48 and 60 h post transfection using the Cell Counting Kit-8 (CCK-8) (Beyotime Institute of Biotechnology, China) following the manufacturer’s instruction. The optical density (OD) 450 nm and 600 nm values were determined by a microplate reader.

Exponentially growing ZF4 cells with 60% confluency were transfected separately with 80 nM miR-125c mimics and mimics-NC. After 24 h, cells were synchronized in G0/G1 phase by serum starvation for 35 h (-FCS) and then released to enter cell cycle progression by adding 10% FCS for another 24 h (+FCS). The -FCS and +FCS cells were collected separately, and fixed in 70% cold ethanol at 4°C overnight. After washing in PBS, the cells were incubated with RNase A for 30min at 37°C and stained with propidium iodide (PI) using the Cell Cycle Detection Kit (KeyGen BioTeck, China) before flow cytometry analysis. Corresponding cell lysates were collected for detection of Cdk2 protein expression.

For the apoptosis analysis, ZF4 cells transfected with miR-125c mimics and mimics-NC were collected at 48 h post transfection, washed in cold PBS, and then subjected to Annexin V-FITC/PI staining for 15 min in dark using the Apoptosis Detection Kit (KeyGen BioTeck). All experiments were performed in triplicate by flow cytometry.

### Quantitative real-time PCR

Total RNA containing miRNA was isolated from zebrafish embryos and ZF4 cells using Trizol reagent (Invitrogen) according to the manufacturer’s instructions. Oligo (dT) and specific stem-loop RT primer (Table 1) were used for cDNA synthesis using Superscript II reverse transcriptase (Takara, China). The quantitative real-time PCR (qRT-PCR) reactions were performed with gene-specific primers (Table 1) using SYBR Green Mix reagent (Takara) on Rotor-Gene Q (Qiagen, Germany). Each experiment was performed in triplicate and the data was analyzed using the 2^–ΔΔCt^ program. The abundance of miRNA and mRNA was normalized to U6 snRNA and 18s rRNA, respectively.

### Western blot analysis

ZF4 cells were lysed in RIPA buffer with 1% PMSF (ComWin Biotech, China). Zebrafish embryos were manually de-yolked and lysed in the lysis buffer. Equal amount of protein samples were separated with 10% SDS-PAGE and transferred to a PVDF membrane (Millipore, USA). The membrane was blocked for 1 h in TBST containing 5% milk at room temperature, followed by 1 h incubation with specific primary antibodies against zebrafish Cdc25a (1:500) (Homemade polyclonal antibody prepared using prokaryotic expression system, affinity-purified and generated in rabbits), Cdk2 (Cyclin-dependent kinase 2, 1:1000) (Aviva Systems Biology, USA), Hif-1α (1:2000) (Homemade polyclonal antibody generated in rabbits) or β-actin (1:500) (Boster, China). The blot was detected with IRDye 800CW anti-rabbit secondary antibody (1:10,000) (Li-Cor Biosciences, USA) and visualized using Odyssey CLx Infrared Imaging System (Li-Cor Biosciences).

### Microinjection, imaging and apoptosis analysis

MiR-125c mimics (Genepharma) were microinjected into 1-cell stages zebrafish embryos at a concentration of 5 µM or 10 µM. For the rescue, miR-125c inhibitor was injected at a concentration of 20 µM or 40 µM were immediately mimics.

Zebrafish embryos at 24, 48, 72 and 96 hpf were anesthetized in tricaine methanesulfonate (MS222, Sigma) and imaged by Leica MZ16FA Microscope using MetaVue software. The heart rate data was attained by means of manual count under the microscope. A total of 145 miR-125c injected embryos and 126 wild-type embryos were counted to get the average heart rate per minute.

To detect apoptotic cell death, terminal transferase dUTP nick end labeling (TUNEL) assay was performed in whole mount zebrafish embryos. Embryos at 48 hfp were manually dechorionated and fixed in 4% paraformaldehyde overnight at 4°C. After washing with PBST and treating with protease K, these embryos were stained using the *In situ* Cell Death Detection Kit, POD (Roche, USA) according to the manufacturer’s instructions. To determine both necrotic and apoptotic cell death, zebrafish embryos at 48 hpf were collected and incubated in 5 µg/mL acridine orange (AO, Sigma) for 20 min. All the embryos were observed and photographed using a fluorescence microscope (Leica M205FA).

### Touch-evoked escape response assay

Wild-type and miR-125c injected embryos were raised in Petri dishes at 28.5°C. Mechanosensory stimuli were delivered to the tail of 3 dpf larvae using an insect pin. Time-lapse images of zebrafish embryos were taken at different time intervals using a Nikon smz1500 microscope with SPOT camera system. The length of time for each fish to leave the frame of view was averaged across fishes (*n* = 6). The larval movements were recorded and monitored using the video tracking mode.

### H.E. staining

Wild-type and miR-125c injected zebrafish larvae were collected at 4 dpf and fixed in Bouin’s solution. After dehydrated in a series of graded ethanol and cleared in xylene, there fish were embedded in paraffin. Then the 5 µm thin serial paraffin sections were cut and mounted on slides and further stained with hematoxylin and eosin (H&E) according to the manufacturer’s instructions.

### Statistical analysis

All analyses were performed in triplicate. Results of qRT-PCR were shown as mean ± SD, other data was represented as mean ± SE. Statistical difference between two groups was evaluated by Student’s *t*-test, with *P* < 0.05 considered statistically significant.

## SUPPLEMENTARY MATERIALS FIGURES AND VIDEOS







## References

[1] Pocock R (2011). Invited review: decoding the microRNA response to hypoxia. Pflugers Arch.

[2] Ren Y, Yeoh KW, Hao P, Kon OL, Sze SK (2016). Irradiation of Epithelial Carcinoma Cells Upregulates Calcium-Binding Proteins That Promote Survival under Hypoxic Conditions. J Proteome Res.

[3] Li M, Xiao D, Zhang J, Qu H, Yang Y, Yan Y, Liu X, Wang J, Liu L, Wang J, Duan X (2016). Expression of LPA2 is associated with poor prognosis in human breast cancer and regulates HIF-1α expression and breast cancer cell growth. Oncol Rep.

[4] Oszajca M, Collet G, Stochel G, Kieda C, Brindell M (2016). Hypoxia-selective inhibition of angiogenesis development by NAMI-A analogues. Biometals.

[5] Burd I, Welling J, Kannan G, Johnston MV (2016). Excitotoxicity as a Common Mechanism for Fetal Neuronal Injury with Hypoxia and Intrauterine Inflammation. Adv Pharmacol.

[6] Schödel J, Grampp S, Maher ER, Moch H, Ratcliffe PJ, Russo P, Mole DR (2016). Hypoxia-inducible Transcription Factors, and Renal Cancer. Eur Urol.

[7] Blick C, Ramachandran A, McCormick R, Wigfield S, Cranston D, Catto J, Harris AL (2015). Identification of a hypoxia-regulated miRNA signature in bladder cancer and a role for miR-145 in hypoxia-dependent apoptosis. Br J Cancer.

[8] Slemc L, Kunej T (2016). Transcription factor HIF1A: downstream targets, associated pathways, polymorphic hypoxia response element (HRE) sites, and initiative for standardization of reporting in scientific literature. Tumour Biol.

[9] Semenza GL (2012). Hypoxia-inducible factors in physiology and medicine. Cell.

[10] McNeill LA, Hewitson KS, Claridge TD, Seibel JF, Horsfall LE, Schofield CJ (2002). Hypoxia-inducible factor asparaginyl hydroxylase (FIH-1) catalyses hydroxylation at the beta-carbon of asparagine-803. Biochem J.

[11] Kaelin WG, Ratcliffe PJ (2008). Oxygen sensing by metazoans: the central role of the HIF hydroxylase pathway. Mol Cell.

[12] Jaakkola P, Mole DR, Tian YM, Wilson MI, Gielbert J, Gaskell SJ, von Kriegsheim A, Hebestreit HF, Mukherji M, Schofield CJ, Maxwell PH, Pugh CW, Ratcliffe PJ (2001). Targeting of HIF-alpha to the von Hippel-Lindau ubiquitylation complex by O2-regulated prolyl hydroxylation. Science.

[13] Jeong JW, Bae MK, Ahn MY, Kim SH, Sohn TK, Bae MH, Yoo MA, Song EJ, Lee KJ, Kim KW (2002). Regulation and destabilization of HIF-1αlpha by ARD1-mediated acetylation. Cell.

[14] Kaluz S, Kaluzová M, Stanbridge EJ (2008). Regulation of gene expression by hypoxia: integration of the HIF-transduced hypoxic signal at the hypoxia-responsive element. Clin Chim Acta.

[15] Papandreou I, Cairns RA, Fontana L, Lim AL, Denko NC (2006). HIF-1 mediates adaptation to hypoxia by actively downregulating mitochondrial oxygen consumption. Cell Metab.

[16] Semenza GL (2003). Targeting HIF-1 for cancer therapy. Nat Rev Cancer.

[17] Nagaraju GP, Bramhachari PV, Raghu G, EI-Rayes BF (2015). Hypoxia inducible factor-1α: Its role in colorectal carcinogenesis and metastasis. Cancer Lett.

[18] Souma T, Nezu M, Nakano D, Yamazaki S, Hirano I, Sekine H, Dan T, Takeda K, Fong GH, Nishiyama A, Ito S, Miyata T, Yamamoto M (2016). Erythropoietin Synthesis in Renal Myofibroblasts Is Restored by Activation of Hypoxia Signaling. J Am Soc Nephrol.

[19] Sarkar K, Rey S, Zhang X, Sebastian R, Marti GP, Fox-Talbot K, Cardona AV, Du J, Tan YS, Liu L, Lay F, Gonzalez FJ, Harmon JW (2012). Tie2-dependent knockout of HIF-1 impairs burn wound vascularization and homing of bone marrow-derived angiogenic cells. Cardiovasc Res.

[20] Dunwoodie SL (2009). The role of hypoxia in development of the mammalian embryo. Dev Cell.

[21] Tomlinson RE, Silva MJ (2015). HIF-1a regulates bone formation after osteogenic mechanical loading. Bone.

[22] Imanirad P, Dzierzak E (2013). Hypoxia and HIFs in regulating the development of the hematopoietic system. Blood Cells Mol Dis.

[23] Lin TY, Chou CF, Chung HY, Chiang CY, Li CH, Wu JL, Lin HJ, Pai TW, Hu CH, Tzou WS (2014). Hypoxiainducible factor 2 alpha is essential for hepatic outgrowth and functions via the regulation of leg1 transcription in the zebrafish embryo. PLoS One.

[24] Loscalzo J (2010). The cellular response to hypoxia: tuning the system with microRNAs. J Clin Invest.

[25] Fabian MR, Sonenberg N, Filipowicz W (2010). Regulation of mRNA translation and stability by microRNAs. Annu Rev Biochem.

[26] Li Z, Meng Q, Pan A, Wu X, Cui J, Wang Y, Li L (2017). MicroRNA-455-3p promotes invasion and migration in triple negative breast cancer by targeting tumor suppressor EI24. Oncotarget.

[27] Nallamshetty S, Chan SY, Loscalzo J (2013). Hypoxia: a master regulator of microRNA biogenesis and activity. Free Radic Biol Med.

[28] Gee HE, Ivan C, Calin GA, Ivan M (2014). HypoxamiRs and cancer: from biology to targeted therapy. Antioxid Redox Signal.

[29] Chen Z, Lai TC, Jan YH, Lin FM, Wang WC, Xiao H, Wang YT, Sun W, Cui X, Li YS, Fang T, Zhao H, Padmanabhan C (2013). Hypoxia-responsive miRNAs target argonaute 1 to promote angiogenesis. J Clin Invest.

[30] Huang J, Peng J, Cao G, Lu S, Liu L, Li Z, Zhou M, Feng M, Shen H (2016). Hypoxia-Induced MicroRNA-429 Promotes Differentiation of MC3T3-E1 Osteoblastic Cells by Mediating ZFPM2 Expression. Cell Physiol Biochem.

[31] Mo J, Zhang D, Yang R (2016). MicroRNA-195 regulates proliferation, migration, angiogenesis and autophagy of endothelial progenitor cells by targeting GABARAPL1. Biosci Rep.

[32] Yang Y, Zhang J, Xia T, Li G, Tian T, Wang M, Wang R, Zhao L, Yang Y, Lan K, Zhou W (2016). MicroRNA-210 promotes cancer angiogenesis by targeting fibroblast growth factor receptor-like 1 in hepatocellular carcinoma. Oncol Rep.

[33] Fasanaro P, D’Alessandra Y, Di Stefano V, Melchionna R, Romani S, Pompilio G, Capogrossi MC, Martelli F (2008). MicroRNA-210 modulates endothelial cell response to hypoxia and inhibits the receptor tyrosine kinase ligand Ephrin-A3. J Biol Chem.

[34] Yang L, Song S, Lv H (2016). MicroRNA-322 protects hypoxia-induced apoptosis in cardiomyocytes via BDNF gene. Am J Transl Res.

[35] Sun X, Zuo H, Liu C, Yang Y (2016). Overexpression of miR-200a protects cardiomyocytes against hypoxia-induced apoptosis by modulating the kelch-like ECH-associated protein 1-nuclear factor erythroid 2-related factor 2 signaling axis. Int J Mol Med.

[36] Yang X, Lei S, Long J, Liu X, Wu Q (2016). MicroRNA-199a-5p inhibits tumor proliferation in melanoma by mediating HIF-1α. Mol Med Rep.

[37] Kinose Y, Sawada K, Nakamura K, Sawada I, Toda A, Nakatsuka E, Hashimoto K, Mabuchi S, Takahashi K, Kurachi H, Lengyel E, Kimura T (2015). The hypoxia-related microRNA miR-199a-3p displays tumor suppressor functions in ovarian carcinoma. Oncotarget.

[38] Yu ZY, Bai YN, Luo LX, Wu H, Zeng Y (2013). Expression of microRNA-150 targeting vascular endothelial growth factor-A is downregulated under hypoxia during liver regeneration. Mol Med Rep.

[39] Tu XM, Gu YL, Ren GQ (2016). miR-125a-3p targetedly regulates GIT1 expression to inhibit osteoblastic proliferation and differentiation. Exp Ther Med.

[40] Yin F, Zhang JN, Wang SW, Zhou CH, Zhao MM, Fan WH, Fan M, Liu S (2015). MiR-125a-3p regulates glioma apoptosis and invasion by regulating Nrg1. PLoS One.

[41] Zhou JN, Zeng Q, Wang HY, Zhang B, Li ST, Nan X, Cao N, Fu CJ, Yan XL, Jia YL, Wang JX, Zhao AH, Li ZW (2015). MicroRNA-125b attenuates epithelial-mesenchymal transitions and targets stem-like liver cancer cells through small mothers against decapentaplegic 2 and 4. Hepatology.

[42] Kim SW, Ramasamy K, Bouamar H, Lin AP, Jiang D, Aguiar RC (2012). MicroRNAs miR-125a and miR-125b constitutively activate the NF-κB pathway by targeting the tumor necrosis factor alpha-induced protein 3 (TNFAIP3, A20). Proc Natl Acad Sci U S A.

[43] Sun YM, Lin KY, Chen YQ (2013). Diverse functions of miR-125 family in different cell contexts. J Hematol Oncol.

[44] Yin H, Sun Y, Wang X, Park J, Zhang Y, Li M, Yin J, Liu Q, Wei M (2015). Progress on the relationship between miR-125 family and tumorigenesis. Exp Cell Res.

[45] Huang CX, Chen N, Wu XJ, Huang CH, He Y, Tang R, Wang WM, Wang HL (2015). The zebrafish miR-462/miR-731 cluster is induced under hypoxic stress via hypoxia-inducible factor 1α and functions in cellular adaptations. FASEB J.

[46] Brown JM (2000). Exploiting the hypoxic cancer cell: mechanisms and therapeutic strategies. Mol Med Today.

[47] Tsai YP, Wu KJ (2012). Hypoxia-regulated target genes implicated in tumor metastasis. J Biomed Sci.

[48] Tse AC, Li JW, Wang SY, Chan TF, Lai KP, Wu RS (2016). Hypoxia alters testicular functions of marine medaka through microRNAs regulation. Aquat Toxicol.

[49] Chan YC, Banerjee J, Choi SY, Sen CK (2012). miR-210: the master hypoxamir. Microcirculation.

[50] Shenoy A, Blelloch RH (2014). Regulation of microRNA function in somatic stem cell proliferation and differentiation. Nat Rev Mol Cell Biol.

[51] Sun CC, Li SJ, Yuan ZP, Li DJ (2016). MicroRNA-346 facilitates cell growth and metastasis, and suppresses cell apoptosis in human non-small cell lung cancer by regulation of XPC/ERK/Snail/E-cadherin pathway. Aging (Albany NY).

[52] Bousquet M, Nguyen D, Chen C, Shields L, Lodish HF (2012). MicroRNA-125b transforms myeloid cell lines by repressing multiple mRNA. Haematologica.

[53] Taylor MA, Parvani JG, Schiemann WP (2010). The pathophysiology of epithelial-mesenchymal transition induced by transforming growth factor-beta in normal and malignant mammary epithelial cells. J Mammary Gland Biol Neoplasia.

[54] Tong Z, Liu N, Lin L, Guo X, Yang D, Zhang Q (2015). miR-125a-5p inhibits cell proliferation and induces apoptosis in colon cancer via targeting BCL2, BCL2L12 and MCL1. Biomed Pharmacother.

[55] Wang X, Ha T, Zou J, Ren D, Liu L, Zhang X, Kalbfleisch J, Gao X, Williams D, Li C (2014). MicroRNA-125b protects against myocardial ischaemia/reperfusion injury via targeting p53-mediated apoptotic signaling and TRAF6. Cardiovasc Res.

[56] Leotta M, Biamonte L, Raimondi L, Ronchetti D, Di Martino MT, Botta C, Leone E, Pitari MR, Neri A, Giordano A, Tagliaferri P, Amodio N (2014). A p53-dependent tumor suppressor network is induced by selective miR-125a-5p inhibition in multiple myeloma cells. J Cell Physiol.

[57] Hammer S, To KK, Yoo YG, Koshiji M, Huang LE (2007). Hypoxic suppression of the cell cycle gene CDC25A in tumor cells. Cell Cycle.

[58] Fernandez-Vidal A, Mazars A, Manenti S (2008). CDC25A: a rebel within the CDC25 phosphatases family?. Anticancer Agents Med Chem.

[59] Shen T, Huang S (2012). The role of Cdc25A in the regulation of cell proliferation and apoptosis. Anticancer Agents Med Chem.

[60] Bertero T, Gastaldi C, Bourget-Ponzio I, Mari B, Meneguzzi G, Barbry P, Ponzio G, Rezzonico R (2013). CDC25A targeting by miR-483–3p decreases CCND-CDK4/6 assembly and contributes to cell cycle arrest. Cell Death Differ.

[61] Le MT, Teh C, Shyh-Chang N, Xie H, Zhou B, Korzh V, Lodish HF, Lim B (2009). MicroRNA-125b is a novel negative regulator of p53. Genes Dev.

[62] Harper JW, Adami GR, Wei N, Keyomarsi K, Elledge SJ (1993). The p21 Cdk-interacting protein Cip1 is a potent inhibitor of G1 cyclin-dependent kinases. Cell.

[63] Giraldez AJ, Cinalli RM, Glasner ME, Enright AJ, Thomson JM, Baskerville S, Hammond SM, Bartel DP, Schier AF (2005). MicroRNAs regulate brain morphogenesis in zebrafish. Science.

[64] Franzosa JA, Bugel SM, Tal TL, La Du JK, Tilton SC, Waters KM, Tanguay RL (2013). Retinoic acid-dependent regulation of miR-19 expression elicits vertebrate axis defects. FASEB J.

[65] Benz A, Kossack M, Auth D, Seyler C, Zitron E, Juergensen L, Katus HA, Hassel D (2016). miR-19b Regulates Ventricular Action Potential Duration in Zebrafish. Sci Rep.

[66] Takacs CM, Giraldez AJ (2016). miR-430 regulates oriented cell division during neural tube development in zebrafish. Dev Biol.

[67] Bhattacharya M, Sharma AR, Sharma G, Patra BC, Nam JS, Chakraborty C, Lee SS (2017). The crucial role and regulations of miRNAs in zebrafish development. Protoplasma.

[68] Gupta V, Discenza M, Guyon JR, Kunkel LM (2012). Beggs AH.α-Actinin-2 deficiency results in sarcomeric defects in zebrafish that cannot be rescued by α-actinin-3 revealing functional differences between sarcomeric isoforms. FASEB J.

[69] Bouldin CM, Snelson CD, Farr GH, Kimelman D (2014). Restricted expression of cdc25a in the tailbud is essential for formation of the zebrafish posterior body. Genes Dev.

[70] Imanirad P, Dzierzak E (2013). Hypoxia and HIFs in regulating the development of the hematopoietic system. Blood Cells Mol Dis.

[71] Wang Y, Wan C, Deng L, Liu X, Cao X, Gilbert SR, Bouxsein ML, Faugere MC, Guldberg RE, Gerstenfeld LC, Haase VH, Johnson RS, Schipani E (2007). The hypoxia-inducible factor alpha pathway couples angiogenesis to osteogenesis during skeletal development. J Clin Invest.

[72] Lin TY, Chou CF, Chung HY, Chiang CY, Li CH, Wu JL, Lin HJ, Pai TW, Hu CH, Tzou WS (2014). Hypoxiainducible factor 2 alpha is essential for hepatic outgrowth and functions via the regulation of leg1 transcription in the zebrafish embryo. PLoS One.

[73] Tomlinson RE, Silva MJ (2015). HIF-1α regulates bone formation after osteogenic mechanical loading. Bone.

[74] Nogare DE, Arguello A, Sazer S, Lane ME (2007). Zebrafish cdc25a is expressed during early development and limiting for post-blastoderm cell cycle progression. Dev Dyn.

[75] Kimmel CB, Ballard WW, Kimmel SR, Ullmann B, Schilling TF (1995). Stages of embryonic development of the zebrafish. Dev Dyn.

[76] Mathelier A, Zhao X, Zhang AW, Parcy F, Worsley-Hunt R, Arenillas DJ, Buchman S, Chen CY, Chou A, Ienasescu H, Lim J, Shyr C, Tan G (2014). JASPAR 2014: an extensively expanded and updated open-access database of transcription factor binding profiles. Nucleic Acids Res.

