# Supplementary material for: The zebrafish miR-125c is induced under hypoxic stress via hypoxia-inducible factor 1α and functions in cellular adaptations and embryogenesis
Source: Oncotarget. 2017 May 18;8(43):73846–59. doi: 10.18632/oncotarget.17994 (PMC5650306; doi:10.18632/oncotarget.17994)
Supplement: Supplementary file 1 [file oncotarget-08-73846-s001.pdf]

# The zebrafish miR-125c is induced under hypoxic stress *via* hypoxia-inducible factor 1α and functions in cellular adaptations and embryogenesis

## SUPPLEMENTARY MATERIALS

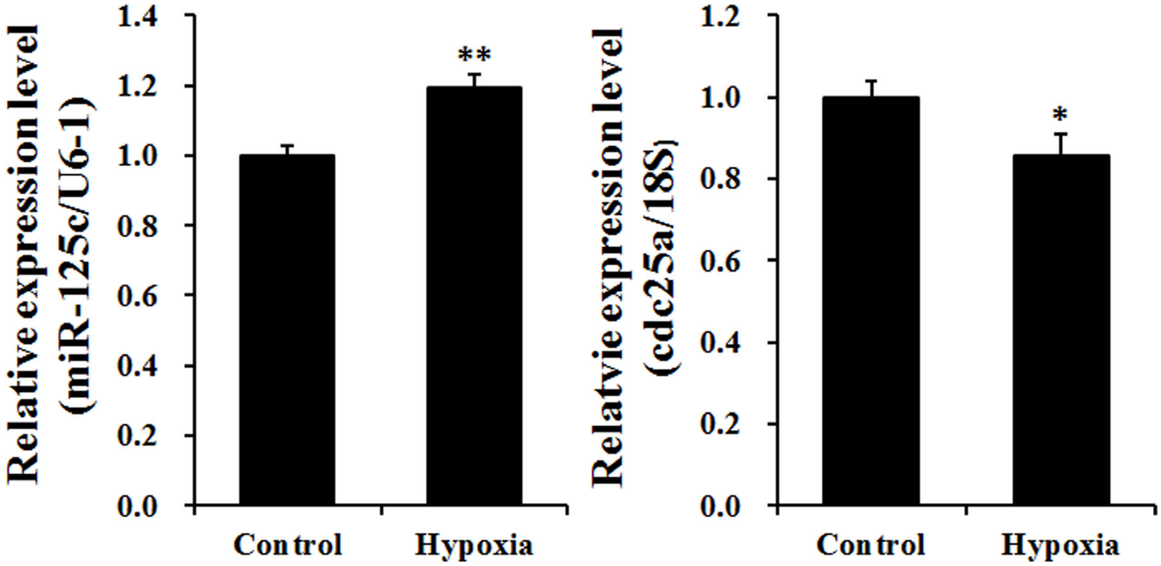


**Supplementary Figure 1: Expression levels of miR-125c and cdc25a in zebrafish embryos in respond to acute hypoxic stress evaluated by qRT-PCR.** Zebrafish embryos were exposed to low oxygen (1.0 mg/L) from 34 hpf to 36 hpf and collected for qRT-PCR analysis. U6-1 and 18s rRNA are used as the endogenous control. Values represent means ± S.D. ($n = 3$, $^{**}P < 0.01$, $^{*}P < 0.05$).

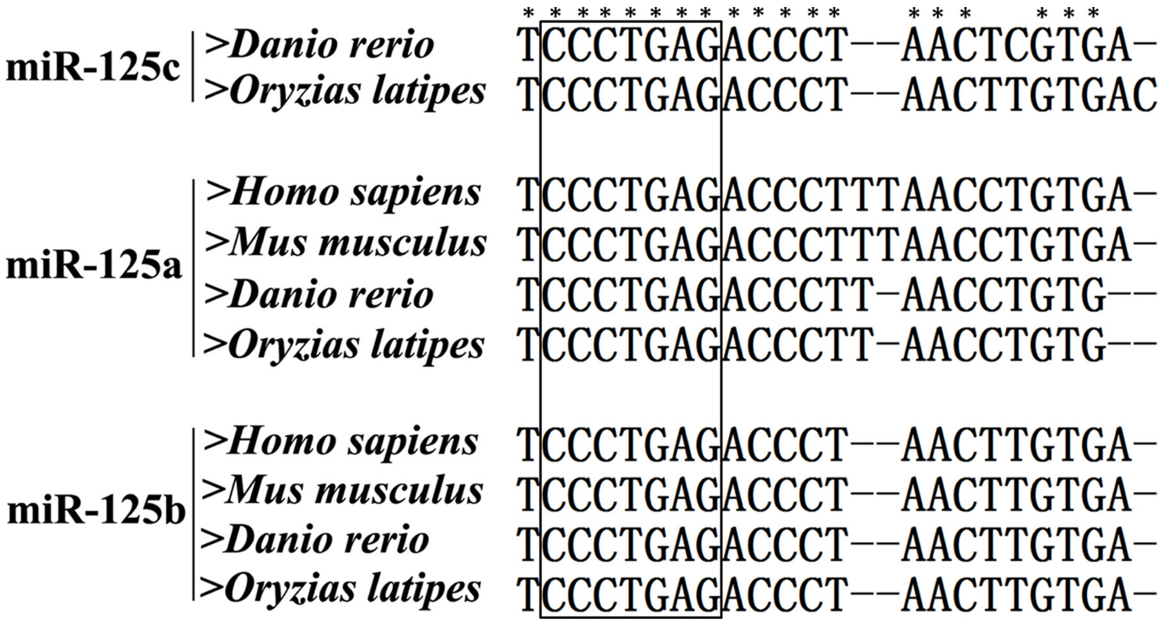


**Supplementary Figure 2: Multiple sequence alignment of miR-125 family.** The seed sequences inside the box are absolutely identical between zebrafish, human and mouse.

**Supplementary Video 1: Impaired motor function.** See Supplementary_Video_1.

**Supplementary Video 2: Normal motor function.** See Supplementary_Video_2.
